# Walking on Collaterals: Unveiling Discrepancies in Gender-Based Variances in Peripheral Arterial Disease

**DOI:** 10.7759/cureus.80337

**Published:** 2025-03-10

**Authors:** Maria A Rodriguez-Santiago, Andres Garcia-Berrios, Jose Martinez-Toro, Marcel Mesa-Pabón

**Affiliations:** 1 Department of Cardiology, University of Puerto Rico, Medical Sciences Campus, San Juan, PRI; 2 Division of Cardiovascular Medicine, University of Puerto Rico, School of Medicine, San Juan, PRI

**Keywords:** ankle-brachial index, gender-specific risk factors, healthcare-system inequalities, peripheral arterial disease, women’s health

## Abstract

A case of a 63-year-old woman with hypertension, chronic kidney disease, and osteoporosis who presented with acute coronary syndrome and new-onset heart failure. Angiography revealed a completely obstructed abdominal aorta, with no circulation to the femoral arteries and perfusion only through collateral vessels. This severe peripheral arterial disease (PAD) was diagnosed during her hospitalization, raising the question of why it had remained undiagnosed for years and whether earlier detection could have improved her outcome. Women with PAD often present with atypical or absent symptoms, leading to underdiagnosis. Early screening with the Ankle-Brachial Index (ABI) is crucial for accurate diagnosis. Moreover, gaps in PAD recognition led to inadequate treatment of comorbidities, less aggressive pharmacologic therapies, and fewer revascularization strategies, resulting in poorer outcomes. This case highlights the unique risk factors and diagnostic challenges in women with PAD, which contribute to healthcare disparities.

## Introduction

Peripheral arterial disease (PAD) is a common cardiovascular condition that causes atherosclerotic and thrombotic disease in the lower extremities, affecting the aortoiliac, femoropopliteal, and infra-popliteal segments [1]. It is often underdiagnosed in women, mostly because of gender bias related to lower screening rates and poor management of frequently encountered risk factors [2]. Women tend to present with PAD at least 10 years older than men, often with more advanced disease [1]. Also, they have a higher prevalence of PAD after age 85 (39%) vs. men (27%) based on the Ankle-Brachial Index (ABI) [2]. Integrating the ABI into routine evaluations can help with early detection and risk prediction [2]. A normal resting ABI ranges from 1.00 to 1.40. An ABI of ≤0.90 is considered abnormal, 0.91-0.99 is borderline, and >1.40 is considered noncompressible [1]. The ABI has limitations, including false positives in patients with medial arterial calcification, such as diabetics or patients with chronic kidney disease [1].

Despite the usefulness of the ABI in diagnosing PAD, current guidelines do not recommend screening for asymptomatic patients, leaving many at risk undiagnosed [1]. To improve detection, we should consider utilizing the Class 2a indication for screening high-risk groups, which should include not only patients with atherosclerosis risk factors, chronic kidney disease (CKD), and diabetes, but also women with pregnancy complications, autoimmune disorders, osteoporosis, and those using hormonal oral contraceptives (OCPs), as each of these factors has been individually associated with PAD.

Current PAD guidelines do not recommend screening for low-risk asymptomatic patients because the likelihood of disease in this population is considered low, but that is our argument.

It is crucial to address gender disparities in the treatment of PAD, such as lower rates of guideline-directed medical therapy (GDMT) and less effective exercise rehabilitation [3]. Increasing women's representation in clinical trials is also essential to improve outcomes, as they currently make up only 33% of participants in PAD intervention studies [3]. Additionally, women with PAD often experience lower revascularization rates and worse post-surgical outcomes. This may be due to factors such as women being diagnosed at an older age, with more advanced disease, and having smaller vessels, which can complicate surgical procedures [4,5].

This case involves a 63-year-old female with a completely occluded abdominal aorta, highlighting how early identification and management of peripheral artery disease (PAD) could have improved her outcomes from major adverse cardiovascular events (MACE).

This article was previously presented as a poster at the 2024 ACC American College of Cardiology Annual Scientific Meeting in April 2024.

## Case presentation

A case of 63-year-old woman with a medical history of hypertension (HTN), chronic kidney disease (CKD), and osteoporosis presented to the hospital with shortness of breath, dyspnea on exertion, and bilateral lower extremity edema of several days' duration. Upon further questioning, she reported experiencing leg weakness and rest pain that had been worsening in intensity over the past five years. Although she had been able to ambulate, she had limited her physical activity during this period. She has been followed by her primary care physician, who recently referred her to a cardiologist for further evaluation of these symptoms.

Her medications included verapamil, atorvastatin, hydrochlorothiazide, and losartan. She was a former smoker with a history of one pack per day for five years. She quit smoking two years ago. Her mother had a history of premature coronary artery disease (CAD).

The patient was initially admitted with pulmonary edema and respiratory distress and was started on IV diuresis and non-invasive respiratory support. On admission, her height was 61 inches, and her weight was 135 lbs. Her BP was BP: 195/107 mmHg, Pulse: 120 bpm, Sat: 90% at room air, and Temp: 36.9. Her physical examination revealed crackles on lung auscultation and a normal heart rate with regular rhythm without murmurs. She presented with well-perfused, warm bilateral lower extremity +1 edema, but there were no palpable pulses in the dorsalis pedis and posterior tibialis.

**Figure 1:**
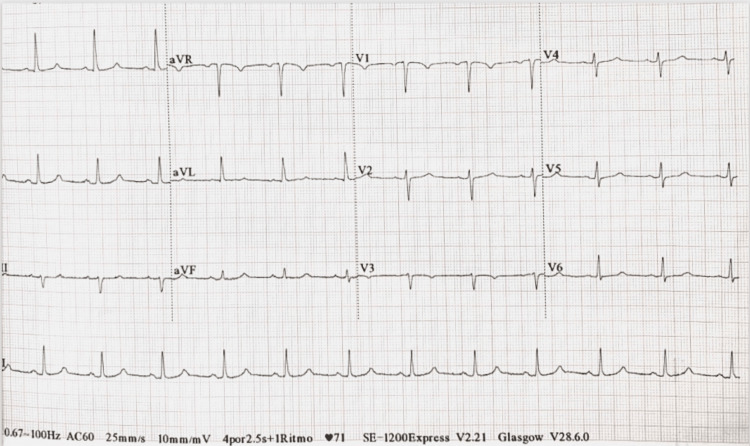
Electrocardiogram Electrocardiogram showing a normal sinus rhythm.

**Figure 2:**
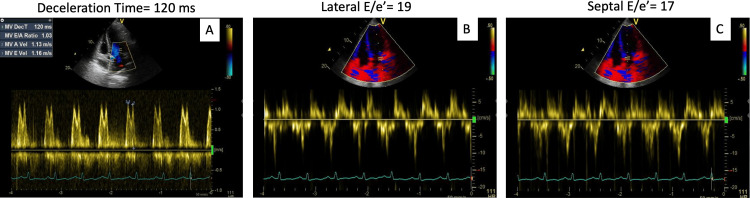
Echocardiogram, apical 4-chamber view with diastolic evaluation Echocardiogram Apical 4 chamber view with pulse wave tissue Doppler imaging showing evidence of elevated left ventricular filling pressures, diagnostic of congestive heart failure. A) Mitral inflow pattern with a deceleration time. B) Lateral E’ velocity C) Septal E’ velocity

Her laboratory results demonstrated a significant rise in NT-proBNP to 2,230 pg/mL, a mild increase in troponin from 0.09 ng/mL upon arrival to 1.91 ng/mL at 3 hours, and evidence of acute kidney injury with a creatinine level of 2.67 mg/dL. Her electrocardiogram showed normal sinus rhythm without evidence of ischemia (Figure 1). Her echocardiogram showed a severely reduced systolic function with an ejection fraction of 30-35% and multiple apical wall motion abnormalities, especially in the anterior wall. Additionally, there was evidence of elevated left ventricular filling pressures, indicating volume overload due to congestive heart failure (Figure 2). She was diagnosed with non-ST elevation acute coronary syndrome (NSTE-ACS) and started on medical management. At that time, the differential diagnosis also included stress-induced cardiomyopathy. Over the next two days, the patient became unresponsive to diuresis and developed anuria with worsening renal function, continuing to experience volume overload that required hemodialysis. Over the following days, she developed hypotensive episodes and was found to have gram-positive bacteremia. She required intravenous antibiotics and vasopressors. A transesophageal echocardiogram (TEE) was performed, which ruled out infective endocarditis. After medical stabilization following a complicated hospitalization, invasive risk stratification was performed.

**Figure 3:**
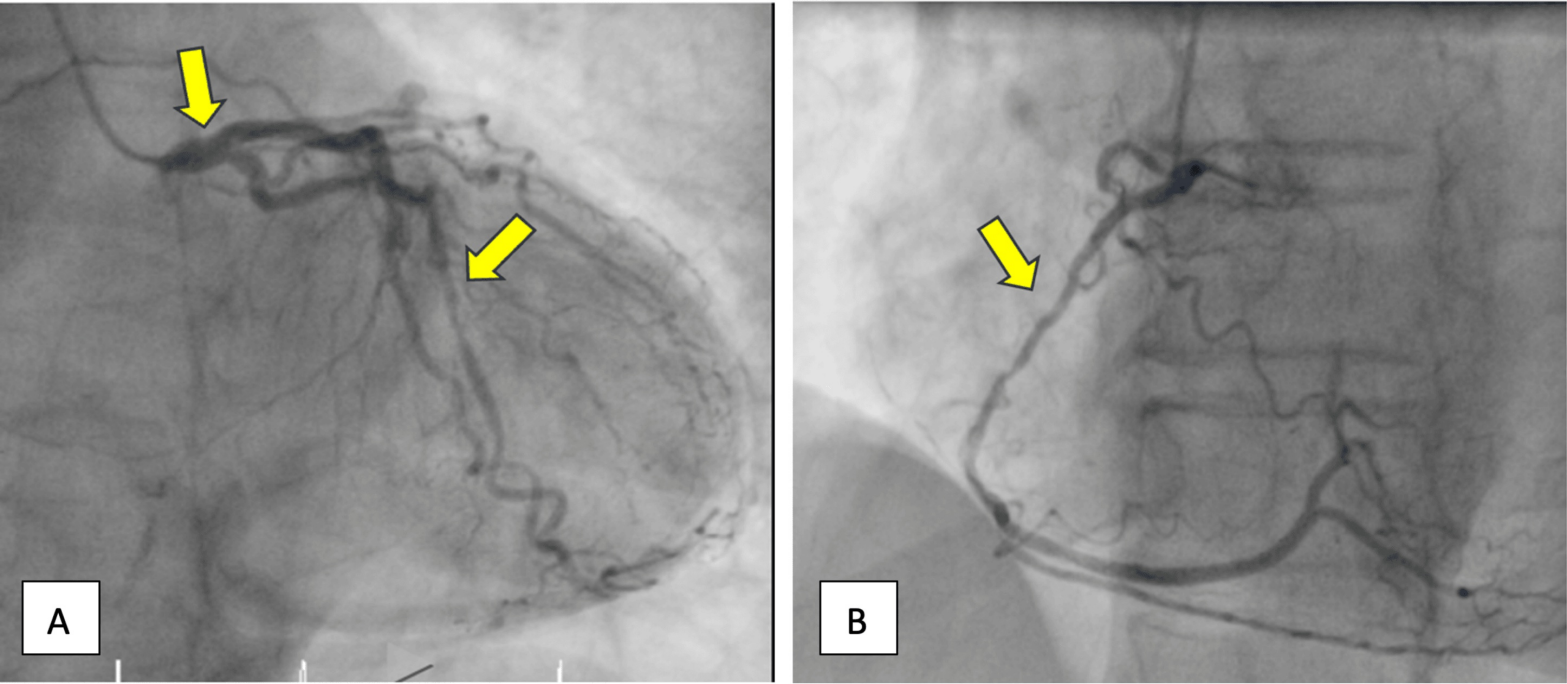
Coronary angiography A) Coronary angiography demonstrating 60% stenosis in the left main and 70% stenosis in the mid-left circumflex. B) Mid-right coronary artery with 90% occlusion (arrows).

**Figure 4:**
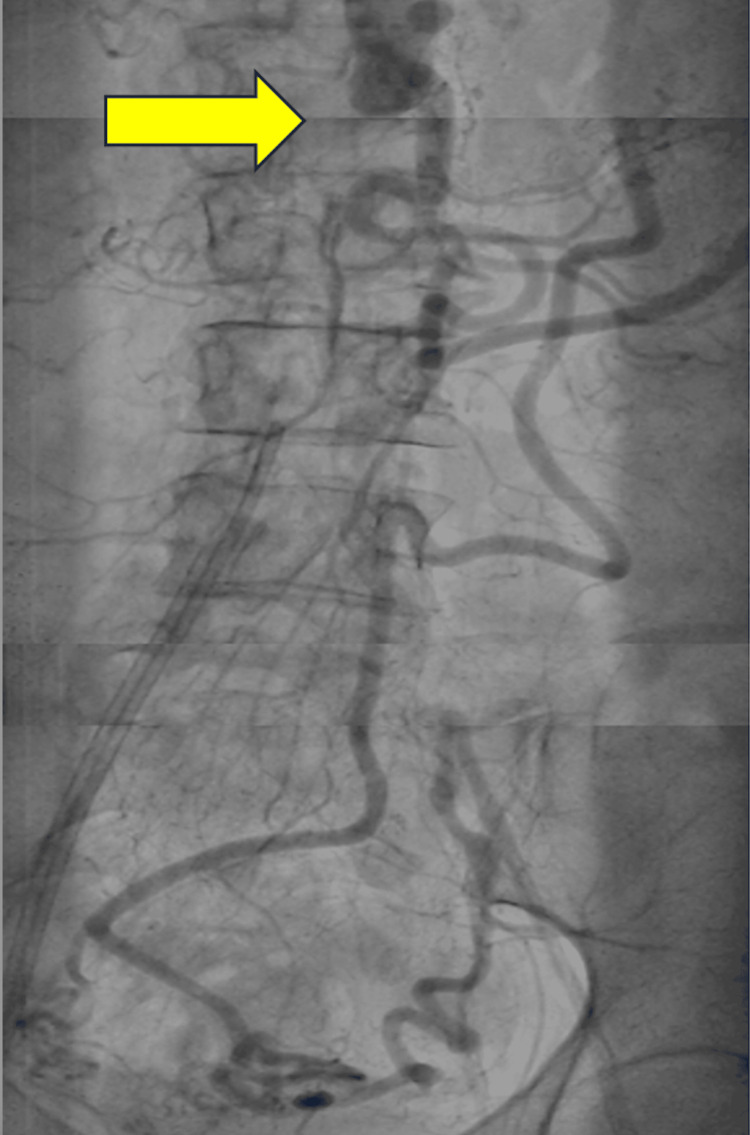
Angiography of the abdominal aorta Angiography demonstrating complete occlusion of the abdominal aorta (yellow arrow). Below the arrow, there is no visible abdominal aorta, but multiple pelvic collateral blood vessels are present.

Her angiography was initially attempted bilaterally via the femoral approach, based on the operator's preference; however, access could not be obtained. Given these difficulties, a right brachial approach was successfully performed. Angiography revealed severe CAD, including a left main stenosis with 60% occlusion, a mid-left circumflex with 70% occlusion, and a mid-right coronary artery with 90% occlusion (Figure 3). Surprisingly, the angiography revealed a complete occlusion of the abdominal aorta with multiple pelvic collateral vessels (Figure 4, Video 1). While the presence of pelvic collateral circulation suggested some adaptive mechanism to preserve perfusion, the full impact of the aortic occlusion on overall circulation remained unclear. This could help explain the poor renal recovery and the paradoxical absence of pulses despite warm extremities.

**Video 1:** Angiography of the abdominal aorta Angiography of the abdominal aorta showing complete occlusion and collaterals.

Due to the patient's multiple comorbidities, including frailty, poor nutrition, heart failure, recently resolved septic shock, and hemodialysis dependence, along with significant anatomic challenges that made surgical intervention risky, the Heart Team decided that continuing medical therapy was the most appropriate option. After completing IV antibiotics, achieving negative blood cultures, and having a temporary hemodialysis catheter placed, the patient was discharged home with ongoing medical management. Long-term dialysis was arranged, and a follow-up with interventional cardiology was scheduled to discuss the benefits and risks of revascularization.

## Discussion

This alarming finding in the abdominal aorta made us wonder why PAD was not diagnosed in all these years and if identifying PAD in this female would have improved her outcome.

Women with PAD face an increased risk for all-cause mortality when compared with women without PAD [6]. Additionally, women with PAD are more likely to have angiographically obstructed CAD [6]. Once women are diagnosed with intermittent claudication, they have more functionally dependent lifestyles, experiencing a two-fold higher mortality rate compared to men [7]. Furthermore, women with PAD often present with multilevel arterial disease at diagnosis [3]. Studies have also shown that women with PAD have a higher relative risk of all-cause mortality compared to women without PAD [6]. Finally, women experience a higher burden of depressive symptoms than their male counterparts, which is significant because these symptoms are associated with more pronounced annual declines due to a greater risk of recurrence and debilitating symptoms [3,8]. These findings highlight the complex gender-specific risk factors that exacerbate the cardiovascular risk in women with PAD, emphasizing the need for a holistic approach to screening and management.

Women with PAD often present with atypical or no symptoms, which leads to misdiagnosis. Atypical symptoms are defined as symptoms that vary from intermittent claudication [5]. These atypical symptoms could be attributed to greater ambulatory limitations resulting from reduced calf muscle hemoglobin oxygen saturation, leading to decreased leg strength [9]. Healthcare providers may erroneously diagnose leg pain secondary to musculoskeletal issues, especially in women with conditions like osteoporosis or osteoarthritis. However, it is essential to know that women are more likely to experience atypical leg symptoms and less frequently report intermittent claudication [3]. Additionally, women with PAD tend to be recognized with fewer cardiovascular risk factors, leading to less aggressive management [1]. McDermott et al. (2000) found that in active women, the severity of PAD, as defined by the ABI, correlated with exertional leg pain, which was not the case in less active women. In less active women, defined as those who walked < 4 blocks weekly, there was no linear relationship between ABI and exertional leg pain [10]. This highlights that the absence of symptoms and a "normal ABI" should not be equated with benign PAD, particularly in less active women. Increased awareness and the use of screening tools, such as the ABI, could improve early diagnosis. Primary care physicians, who often rely on patient history, should consider a patient’s activity level when evaluating PAD risk, as the lack of symptoms may reflect insufficient physical activity rather than the absence of disease.

Healthcare providers can improve the management of women with PAD by starting with a comprehensive approach that takes into consideration all cardiovascular risk factors, including HTN, diabetes, CKD, and a history of smoking, which should prompt early PAD screening [2]. Research indicates that women with PAD are less likely to seek and receive pharmacologic treatment for risk factor reduction when compared to men [11]. Even after revascularization, women are less likely to be discharged on GDMT, which is thought to be due to concerns about frailty, bleeding risk, and the use of antithrombotic medications, as they tend to be older at the time of PAD diagnosis [11]. Therefore, healthcare providers should be aware and proactive at the time of initiating treatment plans, including early referral for exercise programs. Also, early referral for surgical revascularization is essential. Research has shown that women are typically referred for revascularization at an older age compared to men, and they are less likely to undergo interventions [12]. Unfortunately, the response of women to supervised exercise therapy rehabilitation programs has been shown to be less effective, possibly due to impaired calf muscle oxygen saturation [4].

Several risk factors contribute to PAD in both men and women, including smoking, age, diabetes, HTN, and dyslipidemia [2]. However, some risk factors have a stronger or different impact on women. Abdominal obesity, for instance, is more strongly linked to PAD in women, with waist circumference being a more reliable marker of PAD compared to men [13]. Although the exact relationship is not fully understood, some theories suggest it may be related to sex hormones. After menopause, when estrogen levels decline, women experience changes in fat distribution, shifting towards a more male-like pattern with increased abdominal fat [14].

Pregnancy-related complications like pre-eclampsia, gestational hypertension, placental abruption, and placental infarction also increase PAD risk, with the Child Health and Mortality Prevention Surveillance (CHAMPS) study showing a threefold higher risk in women with a history of these conditions [4]. Women with hypertensive disorders of pregnancy have consistently shown underlying systemic endothelial dysfunction [15]. OCP use is associated with a threefold increased risk of PAD diagnosed by an ABI when compared to non-users [16]. Women with osteoporosis may also be at increased risk for PAD [11]. Studies have shown an association between aortic calcification or carotid atherosclerosis and low bone mass in postmenopausal women. Some research suggests that estrogen deficiency may contribute to this risk, as it has been linked to higher levels of low-density lipoprotein cholesterol [2,17]. Women with autoimmune disorders, which are more common in females, also have an increased risk of PAD, as inflammatory markers promote proatherogenic mechanisms [11,18]. Finally, women with CKD are at higher risk for PAD, with women under 70 having a 50% higher incidence of PAD compared to men [6]. Some hypotheses suggest that, since women have smaller vessel diameters than men, this may lead to hemodynamically significant stenosis with a smaller plaque burden [19]. Also, traditional risk factors like diabetes and HTN also affect women differently, with higher A1c levels and blood pressure contributing to a higher prevalence of severe PAD and a 2-3-fold increased risk of developing symptomatic PAD, respectively [11].

This female patient presented with atypical symptoms, namely leg weakness and rest pain, that had been gradually worsening over the past five years but were not identified or managed in a timely manner until this critical event. She had multiple risk factors that contributed to the progression of her PAD. Her medical history of HTN and CKD are well-established risk factors that likely led to endothelial dysfunction and increased atherosclerotic risk. Additionally, her osteoporosis may have played a role, particularly through associations with aortic calcification. Her history of smoking, although she quit two years ago, likely accelerated the development of atherosclerotic changes. When combined, these factors likely contributed to the significant occlusion of the abdominal aorta, highlighting the need for a more comprehensive approach to managing women with PAD, especially those with multiple comorbidities.

## Conclusions

This case emphasizes the critical need for increased awareness and early screening of PAD in women, particularly those with multiple cardiovascular risk factors such as HTN, CKD, and osteoporosis. As demonstrated by this patient, who reported progressively worsening leg weakness and lower extremity pain over the past five years, the atypical presentation of PAD in women often leads to delayed diagnosis. A high level of suspicion and understanding of gender-specific risk factors is essential to improving the time to diagnosis for women with PAD. We suggest implementing routine ABI screening not only in patients with well-known risk factors such as atherosclerotic disease and diabetes but also in women with poor physical activity, sedentary lifestyles, osteoporosis, CKD, depression, a history of pregnancy complications, past use of oral contraceptives, and those with inflammatory or autoimmune diseases. While increasing representation in clinical trials is crucial, public awareness remains a key first step in improving women’s PAD outcomes. Targeting women-specific risk factor management is recommended, including asking about prior pregnancy history, current physical activity levels, a history of pathological fractures, and mental health disorders, all of which are pertinent for PAD diagnosis. We must also be mindful of potential biases to ensure early referrals for interventions and the timely initiation of antithrombotic therapy, ultimately improving treatment for women with PAD. Early identification of PAD in this patient could have prompted more aggressive treatment, potentially preventing the development of such advanced coronary artery disease.
